# Research proposal: inflammation and oxidative stress in coronary artery bypass surgery graft: comparison between diabetic and non-diabetic patients

**DOI:** 10.1186/s13104-018-3743-5

**Published:** 2018-09-03

**Authors:** Ana Catarina Romano e Silva, Glauber Monteiro Dias, Jorge José de Carvalho, Andrea De Lorenzo, Daniel Arthur Barata Kasal

**Affiliations:** 1 National Institute of Cardiology, Ministry of Health, Rua das Laranjeiras No. 374, Rio de Janeiro, RJ 22240-006 Brazil; 2 Biomedical Center, State University of Rio de Janeiro, Rio de Janeiro, RJ Brazil

**Keywords:** Inflammation, Oxidative stress, Diabetes mellitus, Coronary artery bypass grafting

## Abstract

**Background:**

Diabetes mellitus patients (DM) have more severe progression of atherosclerotic disease than non-diabetic (NDM) individuals. In situ inflammation and oxidative stress are key points in the pathophysiology of atherosclerosis, a concept largely based on animal model research. There are few studies comparing inflammation and oxidative stress parameters in medium-sized arteries between DM and NDM patients. A fragment of the internal mammary artery used in coronary artery bypass grafting (CABG) will be employed for this purpose

**Objective:**

To assess the expression of inflammatory markers tumor necrosis factor-α, transforming growth factor-β1, nuclear factor kappa B, the enzymes superoxide dismutase, and catalase in the vascular wall of the arterial graft used in CABG, comparing DM and NDM patients

**Results:**

The present study will add information to the vascular degenerative processes occurring in diabetic patients.

## Introduction

Coronary artery disease (CAD) is the main cause of death in diabetic patients [1]. Coronary artery bypass grafting (CABG) is one of the most important strategies for CAD treatment [2]. The internal mammary artery (IMA) is the graft of choice for revascularization of the left anterior descending coronary artery [3]. During CABG, not the whole extension of IMA is used by the surgeon. A fragment of the unemployed part of the vessel provides the opportunity to access an artery with similar structure compared to the epicardial coronary arteries [4], as a model for evaluating vascular degenerative processes in these patients.

Increased inflammation, oxidative stress, and the resulting endothelial dysfunction are key factors to the severity of atherosclerosis in diabetes [5]. While research on the field is largely based on animal models [6–8], only few studies have addressed diabetes-induced molecular mechanisms in human medium-sized arteries [9, 10].

The goal of this study is to identify the contribution of diabetes to the vascular expression of a group of molecules in CAD patients. For this purpose, we will compare IMA samples obtained from diabetic (DM) versus non-diabetic (NDM) patients subjected to CABG. The selected inflammatory markers will be tumor necrosis factor-α (TNF-α), transforming growth factor β-1 (TGFβ-1), and nuclear factor kB (NF-κB). Oxidative stress pathways will be evaluated by the expression of the enzymes superoxide dismutase (SOD) and catalase (CAT).

TNF-α induces endothelial expression of cell adhesion molecules, vascular cell adhesion molecule (VCAM) and intercellular cell adhesion molecule (ICAM), both important for the infiltration of monocytes at the intima of the vessel [11]. TGFβ-1 is a multifunctional peptide which stimulates cell proliferation, migration, and extracellular matrix deposition, contributing to the vascular remodeling of atherosclerosis [12]. NF-κB is a transcription factor activated both by hyperglycemia and reactive oxygen species (ROS) [13], regulating the expression of cytokines and adhesion proteins, modulating vascular inflammation, and the recruitment of immune cells to the vascular wall [14, 15]. Superoxide dismutase is an essential component of cell defense against ROS, converting superoxide anion to hydrogen peroxide. Catalase is an enzyme with complementary action in ROS elimination, converting hydrogen peroxide to water [16]. An excess of ROS in the vascular tissue is directly related to endothelial dysfunction [17].

## Main text

### Methods

#### Study design and patients

This will be a cross-sectional study with convenience sampling. Individuals will be consecutive male adult patients admitted for elective CABG. The surgical strategy will be defined by the National Institute of Cardiology heart team, with none of its members directly involved in the study. Eligibility criteria are male individuals above 18 years old and IMA use in CABG. Exclusion criteria are kidney failure with hemodialysis, known acute or chronic infectious disease, presence of autoimmune disease, or use of immunosuppressants. The study is approved by the Local Ethics Research Committee under protocol # 33705614.2.0000.5272, and informed consent will be obtained.

Based on previous studies with vascular tissue obtained from CABG [9, 10], the total estimated number of patients necessary for this study is 50, equally divided in DM and NDM.

#### Study variables

Data from the medical records will be obtained: anthropometric data (height, body weight, abdominal circumference, and body mass index), associated diseases, social habits (cigarette and ethanol consumption), family history of cardiovascular disease, medications, presurgical transthoracic echocardiogram (ejection fraction by Teicholz method), coronary angiography (number of stenotic vessels and percent luminal stenosis), and plasma biochemistry (fasting glucose, sodium, potassium, blood urea nitrogen, creatinine, low-density lipoprotein, high-density lipoprotein, and triglycerides).

#### Vessel collection and processing

The evaluation of the selected markers will be performed by quantitative real time-polymerase chain reaction (qPCR) and immunohistochemistry. Initially, the unused fragment of IMA during CABG will be harvested by the surgeon in a glass tube containing 25 ml of cold, sterilized, phosphate buffered saline (PBS). Immediately after collection, the fragment will be delicately flushed and carefully dissected for adventitia removal. It will be divided in two parts. One will be placed in fixative solution paraformaldehyde 4% in PBS. The other portion will be weighed and frozen in liquid nitrogen, for mechanical maceration until pulverization for RNA purification.

#### Real-time polymerase chain reaction

Total RNA extraction from pulverized tissue will be performed with an extraction kit (mirVana^®^, Life Technologies, Carlsbad, California, USA). The protocol will be followed according to the manufacturer instructions. The resulting RNA fraction will be quantified by the use of 260 nm spectrophotometry. High Capacity^®^ kit (Life Technologies, Carlsbad, California, USA) will be employed for reverse transcriptase PCR, of an aliquot of total RNA (2 ng/μl) diluted in ultrapure water treated with diethylpyrocarbonate (DEPC). Thermocycler (Biocycler) setting will be 25 °C for 10 min, followed by 37 °C for 120 min and 85 °C for 5 s, as recommended by the kit. Complementary DNA (cDNA) will be frozen at − 20 °C for posterior utilization. Glyceraldehyde 3-phosphate dehydrogenase (GAPDH) will be the house-keeping gene. The primers to be used in the study are listed in Table 1, based on literature research [18–20].

**Table 1:** Nucleotide sequence of the primers to be used in the study

		Amplicon size (bp)
SOD2 F:	5′-CTG GAC AAA CCT CAG CCC TA-3′	62
SOD2 R:	5′-TGA TGG CTT CCA GCA ACT C-3′	
CAT F:	5′-AGT GAT CGG GGG ATT CCA GA-3′	159
CAT R:	5′-AAG TCT CGC CGC ATC TTC AA-3′	
TNF-α F:	5′-GTT CCT CAG CCT CTT CTC CT-3′	186
TNF-α R:	5′-ACA ACA TGG GCT ACA GGC TT-3′	
TGF-β1 F:	5′-TGA ACC GGC CTT TCC TGC TTC TCA TG-3′	151
TGF-β1 R:	5′-GCG GAA GTC AAT GTA CAG CTG CCG C-3′	
NF-κB F:	5′-AAA CAC TGT GAG GAT GGG ATC TG-3′	64
NF-κB R:	5′-CGA AGC CGA CCA CCA TGT-3′	
GAPDH F:	5′-GAA GGT GAA GGT CGG AGT C-3′	226
GAPDH R:	5′-GAA GAT GGT GAT GGG ATT TC-3′	

*Bp* base pairs, *CAT* catalase, *F* forward, *GADPH* glyceraldehyde 3-phosphate dehydrogenase, *NF-κB* nuclear transcription factor kappa B, *R* reverse, *SOD2* superoxide dismutase-2, *TGF-β1* transforming growth factor β-1, *TNF-α* tumor necrosis factor-α

qPCR quantification will be performed at thermocycler VIIA7 (Applied Biosystems, Foster City, California, USA), with 10 μl of SYBR Green Master Mix (Life Technologies, Carlsbad, California, USA), 2 μl of cDNA, 0.8 μl of primer solution (3 μM), and 7.2 μl of DEPC water. All samples will be analyzed in triplicate. Optimal primer concentration and PCR efficiency will be determined for all markers. The results of the amplification curves will be analyzed with the software available in the thermocycler manufacturer website (http://www.thermofisher.com/br/en/home/cloud.html).

#### Immunohistochemistry

The fragment fixated in paraformaldehyde 4% will be stored under refrigeration until processing with alcohol dehydration, xylol clearing, and paraffin inclusion. Transversal sections 5 μm thick will be obtained for histological analysis. Primary monoclonal antibodies will be used for TNF-α, TGF-β (Santa Cruz Biotechnology, Dallas, USA), and NF-κB (ABCAM, Cambridge, USA), with Histostain Plus 3rd generation detection kit (Invitrogen, Waltham, USA), with biotinylated secondary antibody. The color reaction uses chromogen diaminobenzidine (DAB), and peroxidase as substrate. After immunohistochemistry, the glass slides will be stained with hematoxylin, for nuclei identification. The slides will be photographed and quantified using the software Image Pro-Plus^®^ 4.5 (Image Pro, Dallas, USA). Quantification of the immunologically positive area will be performed from five distinct fields from each section, using 40× magnification. The investigator will be blinded to the group analyzed.

#### Statistical analysis

Clinical and laboratory data will be shown descriptively. Patients will be divided in two main groups, DM and NDM. Variables will be tested for normality using the Kolmogorov–Smirnov test. Comparison of numerical variables between DM and NDM with normal distribution will be analyzed by Student-T test, and variables with non Gaussian distribution will be analyzed by Mann–Whitney test. Categorical variables will be analyzed by Chi Square or Fisher’s exact test. P values < 0.05 will be considered significant. The results of normal distribution variables will be presented as mean ± SD. Non Gaussian variables will be presented as median and percentiles. The statistical package to be used for the statistical analyses is Prism version 6.0 (Graphad Software, La Jolla, CA, USA).

### Discussion

This study will analyze clinical, laboratory, and histological data from CABG patients, divided in two groups, diabetic versus non-diabetic. We will first identify whether there are other clinical differences between the groups, besides the diagnosis of diabetes mellitus and the use of diabetes medications. Previous analysis of the clinical profile of CABG patients at our Institution verified the same level of exposure to all other risk factors for CAD in the two groups, except for diabetes (data not shown).

A previous study has demonstrated an increase of TGF-β1 in vascular tissue from rats with diabetes-induced nephropathy [21]. Prior animal studies have demonstrated by qPCR the increase of CAT and SOD expression in vascular tissues [22, 23] in diabetes. This could represent a tentative compensatory response to increased oxidative stress in the vascular tissue of diabetic patients. In a single study with human tissue, there was no difference in the expression and activity of SOD, when comparing the saphenous vein with IMA from CAD patients [9]. However, this analysis did not address the contribution of diabetes.

In the study of Wildhirt et al. using morphology and histopathology analysis of the radial artery (RA) in CABG, no association between intima thickness and diabetes, hypertension, or smoking was found. However, only four diabetic patients were included [24]. Another study, with a geographically distinct population, found the association between diabetes and media calcification in RA [25].

Preil et al. studied human IMA from CABG, comparing DM and NDM. The authors found an increased deposition of the basement membrane components alpha-1 and alpha-2 type IV collagen, gamma-1 and beta-2 laminin in diabetic patients [10].

Recent studies have identified plasma markers with prognostic properties in CABG patients. Examples are the measurements of endothelial-leukocyte adhesion molecule 1 (ELAM-1), interleukin 6 and 8, and TNF-α [26]. In the future, follow-up of the patients in the present study could eventually lead to the identification of additional prognostic markers, to be obtained from a sample of IMA used in CABG.

## Limitations and strengths of the study

The present study will be performed on a convenience sample from a single healthcare center, and therefore may not be representative of the entire population of CAD patients.

There is no healthy control in the study, as it would not be ethical to obtain IMA samples from individuals not subjected to open-chest CABG. All samples will be obtained from advanced CAD patients, and the relative contribution of diabetes could be more relevant in the early stages of the disease. However, recent work has demonstrated differences in proteomic analysis between diabetic and non-diabetic patients with established CAD [10]. The exclusion of female subjects was chosen, in order to avoid estradiol-induced attenuation of vascular inflammation [27]. While this aims to eliminate a possible source of variability of the results, it implies the study can be translated to phenomena occurring only in human male vascular tissue.

Finally, vascular degeneration and arteriosclerosis affect vessels in heterogeneous fashion across their length. The fragment available for evaluation in this study may not necessarily represent the condition of the entire grafted segment during CABG.

The main strength of the present study is the access to IMA fragments, a distinctive opportunity to obtain human vascular tissue from living individuals, in order to evaluate in fresh material for protein transcription and histological analysis.

## Abbreviations

Bp: base pairs
CABG: coronary artery bypass grafting
CAD: coronary artery disease
CAT: catalase
cDNA: complemmentary DNA
DAB: diaminobenzidine
DM: diabetes mellitus
DEPC: diethylpyrocarbonate
ELAM-1: endothelial-leukocyte adhesion molecule 1
F: forward
GAPDH: glyceraldehyde 3-phosphate dehydrogenase
ICAM: intercellular cell adhesion molecule
IMA: internal mammary artery
NDM: non-diabetic
NF-κB: nuclear transcription factor κB
PBS: phosphate buffered saline
qPCR: real time-polymerase chain reaction
R: reverse
RA: radial artery
RNA: ribonucleic acid
ROS: reactive oxygen species
SD: standard deviation
SOD: superoxide dismutase
TGFβ-1: transforming growth factor β-1
TNF-α: tumor necrosis factor-α
VCAM: vascular cell adhesion molecule
